# Supplementary material for: Exploring the Microdiversity Within Marine Bacterial Taxa: Toward an Integrated Biogeography in the Southern Ocean
Source: Front Microbiol. 2021 Jul 14;12:703792. doi: 10.3389/fmicb.2021.703792 (PMC8317501; doi:10.3389/fmicb.2021.703792)
Supplement: Supplementary File 1 — Pairwise PERMANOVA on Spirochaeta OTUs composition dissimilarities among localities. p-values are adjusted using the default Bonferroni method implemented in the pairwiseAdonis R package and are considered as significant < 0.05. [file Data_Sheet_1.zip › Supplementary Material 2.PPTX]

## Slide 1
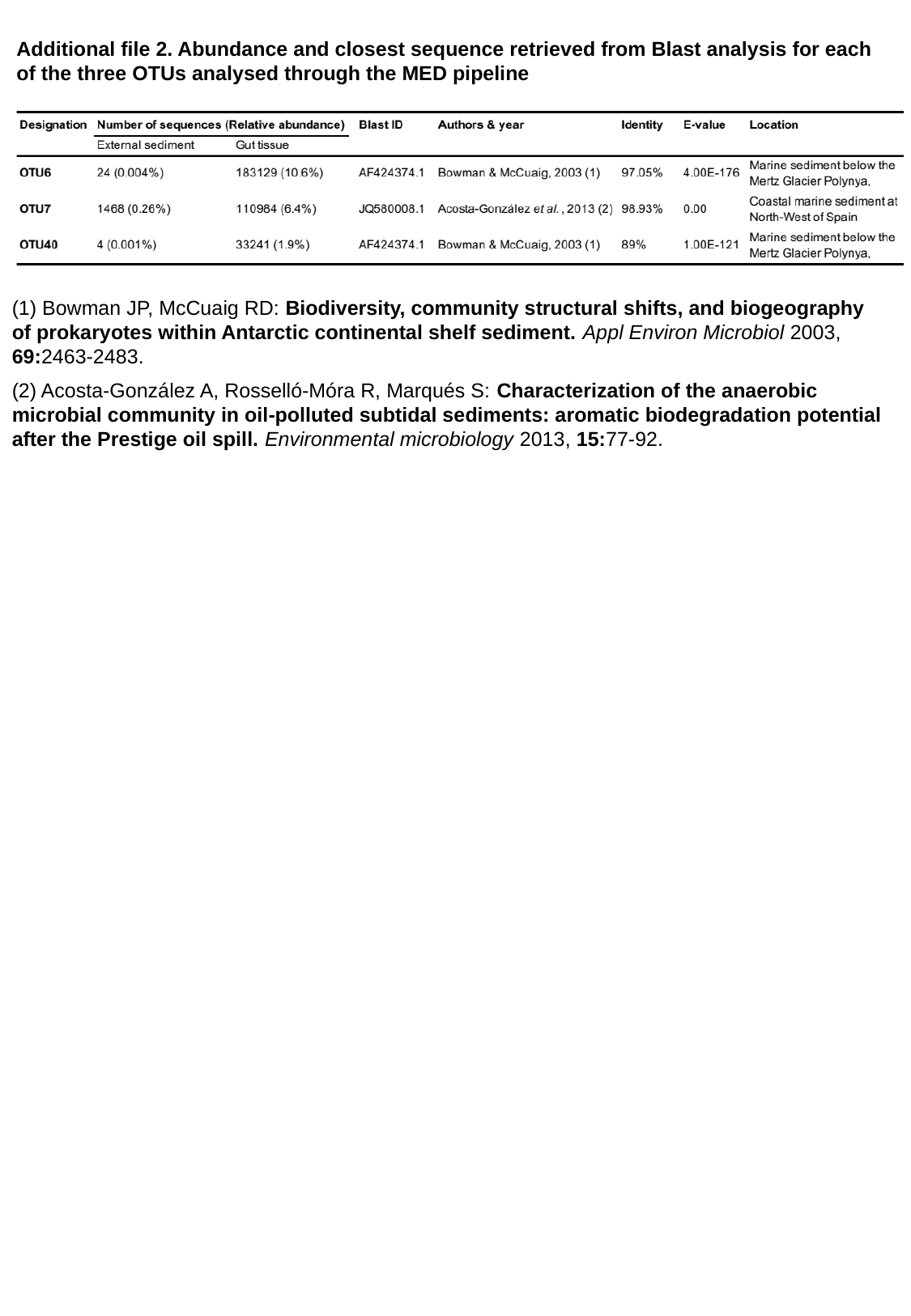

Additional file 2. Abundance and closest sequence retrieved from Blast analysis for each of the three OTUs analysed through the MED pipeline
(1) Bowman JP, McCuaig RD: Biodiversity, community structural shifts, and biogeography of prokaryotes within Antarctic continental shelf sediment. Appl Environ Microbiol 2003, 69:2463-2483.
(2) Acosta‐González A, Rosselló‐Móra R, Marqués S: Characterization of the anaerobic microbial community in oil‐polluted subtidal sediments: aromatic biodegradation potential after the Prestige oil spill. Environmental microbiology 2013, 15:77-92.
