# Supplementary material for: Exploring the Microdiversity Within Marine Bacterial Taxa: Toward an Integrated Biogeography in the Southern Ocean
Source: Front Microbiol. 2021 Jul 14;12:703792. doi: 10.3389/fmicb.2021.703792 (PMC8317501; doi:10.3389/fmicb.2021.703792)
Supplement: Supplementary File 1 — Pairwise PERMANOVA on Spirochaeta OTUs composition dissimilarities among localities. p-values are adjusted using the default Bonferroni method implemented in the pairwiseAdonis R package and are considered as significant < 0.05. [file Data_Sheet_1.zip › Supplementary Material 3.PPTX]

## Slide 1
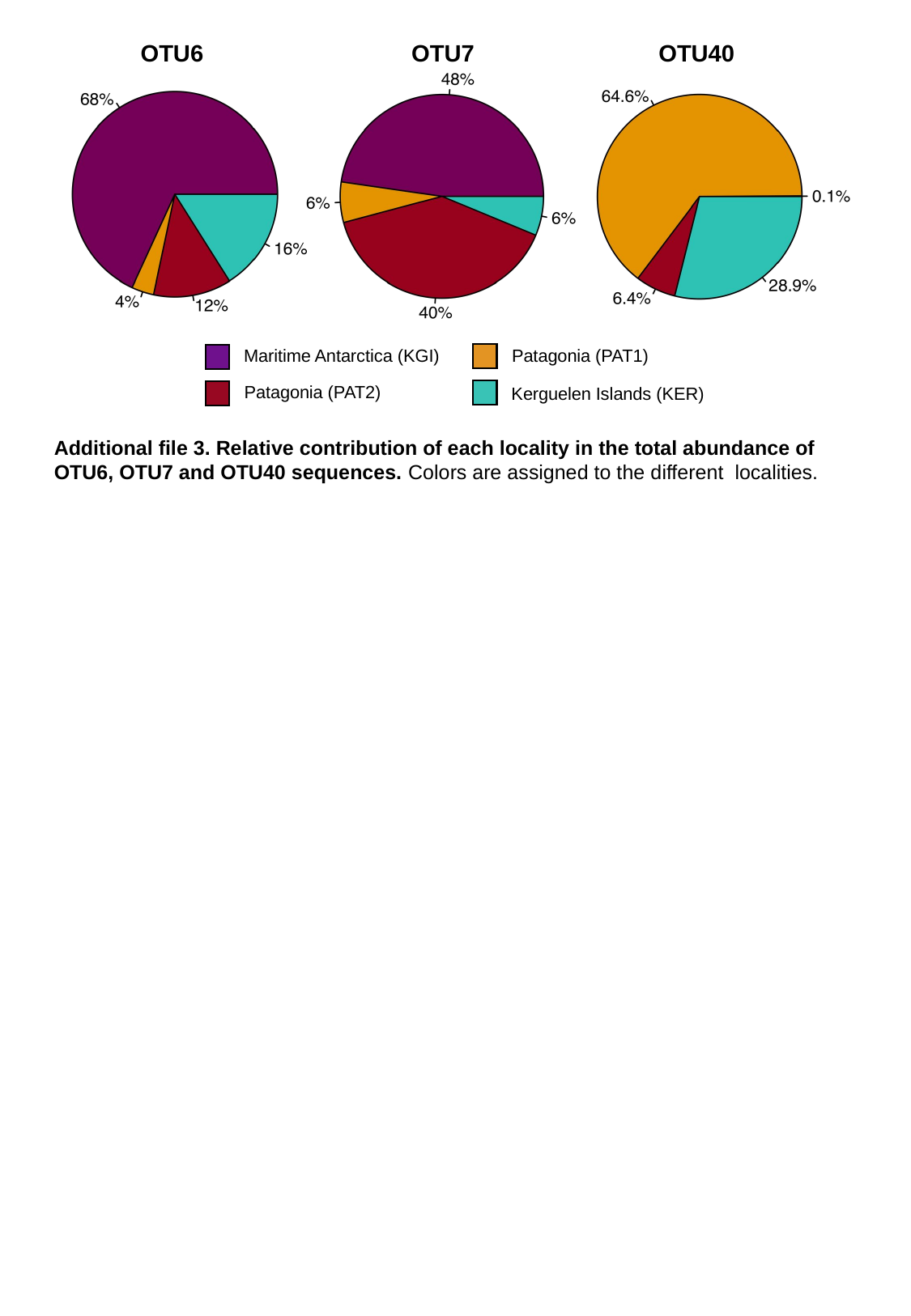

OTU6
OTU7
OTU40
Patagonia (PAT1)
Maritime Antarctica (KGI)
Patagonia (PAT2)
Kerguelen Islands (KER)
Additional file 3. Relative contribution of each locality in the total abundance of OTU6, OTU7 and OTU40 sequences. Colors are assigned to the different localities.
