# Supplementary material for: Exploring the Microdiversity Within Marine Bacterial Taxa: Toward an Integrated Biogeography in the Southern Ocean
Source: Front Microbiol. 2021 Jul 14;12:703792. doi: 10.3389/fmicb.2021.703792 (PMC8317501; doi:10.3389/fmicb.2021.703792)
Supplement: Supplementary File 1 — Pairwise PERMANOVA on Spirochaeta OTUs composition dissimilarities among localities. p-values are adjusted using the default Bonferroni method implemented in the pairwiseAdonis R package and are considered as significant < 0.05. [file Data_Sheet_1.zip › Supplementary Material 4.PPTX]

## Slide 1
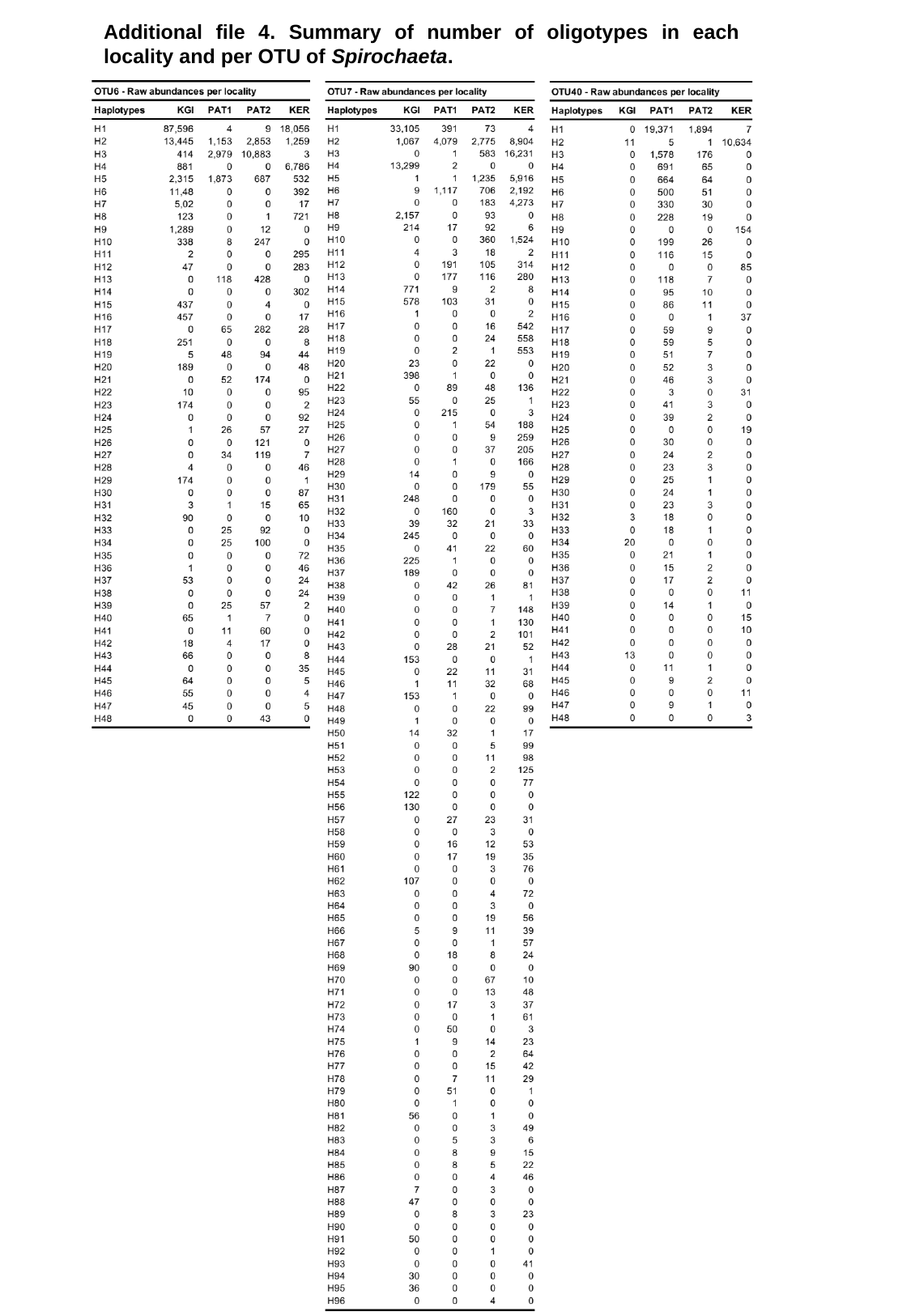

Additional file 4. Summary of number of oligotypes in each locality and per OTU of Spirochaeta.
