# Supplementary material for: Exploring the Microdiversity Within Marine Bacterial Taxa: Toward an Integrated Biogeography in the Southern Ocean
Source: Front Microbiol. 2021 Jul 14;12:703792. doi: 10.3389/fmicb.2021.703792 (PMC8317501; doi:10.3389/fmicb.2021.703792)
Supplement: Supplementary File 1 — Pairwise PERMANOVA on Spirochaeta OTUs composition dissimilarities among localities. p-values are adjusted using the default Bonferroni method implemented in the pairwiseAdonis R package and are considered as significant < 0.05. [file Data_Sheet_1.zip › Supplementary Material 7.PPTX]

## Slide 1
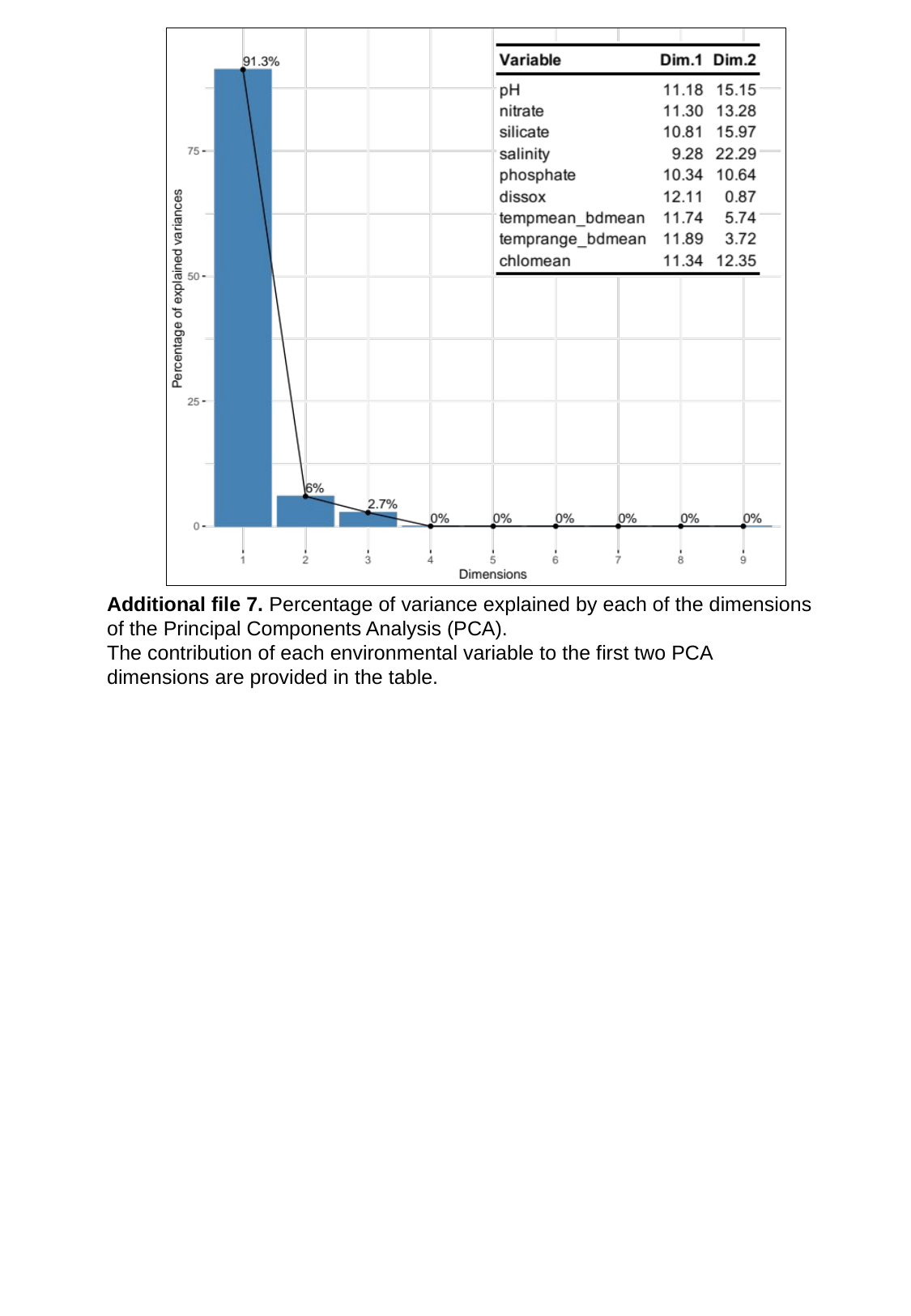

Additional file 7. Percentage of variance explained by each of the dimensions of the Principal Components Analysis (PCA).
The contribution of each environmental variable to the first two PCA dimensions are provided in the table.
